# Blood-Brain Barrier Cellular Responses Toward Organophosphates: Natural Compensatory Processes and Exogenous Interventions to Rescue Barrier Properties

**DOI:** 10.3389/fncel.2018.00359

**Published:** 2018-10-16

**Authors:** Orly Ravid, Shirin Elhaik Goldman, David Macheto, Yael Bresler, Raquel Ines De Oliveira, Sigal Liraz-Zaltsman, Fabien Gosselet, Lucie Dehouck, Michal Schnaider Beeri, Itzik Cooper

**Affiliations:** ^1^The Joseph Sagol Neuroscience Center, Sheba Medical Center, Tel Hashomer, Ramat Gan, Israel; ^2^Interdisciplinary Center Herzliya, Herzliya, Israel; ^3^Blood-Brain Barrier Laboratory (LBHE), Université d’Artois, Lens, France; ^4^Department of Psychiatry, Icahn School of Medicine at Mount Sinai, New York, NY, United States

**Keywords:** blood-brain barrier, organophosphates, paraoxon, tight junction, permeability

## Abstract

Organophosphorus compounds (OPs) are highly toxic chemicals widely used as pesticides (e.g., paraoxon (PX)- the active metabolite of the insecticide parathion) and as chemical warfare nerve agents. Blood-brain barrier (BBB) leakage has been shown in rodents exposed to PX, which is an organophosphate oxon. In this study, we investigated the cellular mechanisms involved in BBB reaction after acute exposure to PX in an established *in vitro* BBB system made of stem-cell derived, human brain-like endothelial cells (BLECs) together with brain pericytes that closely mimic the *in vivo* BBB. Our results show that PX directly affects the BBB *in vitro* both at toxic and non-toxic concentrations by attenuating tight junctional (TJ) protein expression and that only above a certain threshold the paracellular barrier integrity is compromised. Below this threshold, BLECs exhibit a morphological coping mechanism in which they enlarge their cell area thus preventing the formation of meaningful intercellular gaps and maintaining barrier integrity. Importantly, we demonstrate that reversal of the apoptotic cell death induced by PX, by a pan-caspase-inhibitor ZVAD-FMK (ZVAD) can reduce PX-induced cell death and elevate cell area but do not prevent the induced BBB permeability, implying that TJ complex functionality is hindered. This is corroborated by formation of ROS at all toxic concentrations of PX and which are even higher with ZVAD. We suggest that while lower levels of ROS can induce compensating mechanisms, higher PX-induced oxidative stress levels interfere with barrier integrity.

## Introduction

Organophosphorus compounds are highly toxic chemicals widely used as chemical warfare nerve agents (e.g., soman, sarin) and as pesticides (e.g., chlorpyrifos and PX- the active metabolite of parathion). Pesticide poisoning is one of the most common poisonings worldwide, estimated at one million cases each year with several hundred thousand deaths (Pandit et al., 2011). According to the world health organization available data are too limited to estimate the global health impacts of pesticides, however, the global impact of self-poisoning (suicides) from preventable pesticide ingestion has however been estimated to amount to 186,000 deaths and 4,420,000 Disability Adjusted Life Years in 2002. The blood–brain barrier (BBB) is a selective barrier formed by the endothelial cells (ECs) that line cerebral capillaries, together with perivascular elements such as the closely associated astrocytic end-feet processes, perivascular neurons, pericytes and basal lamina (Cecchelli et al., 2007). Physiologically, the BBB protects the brain from the compositional fluctuations of compounds that occur in the circulation and plays a major role in maintaining the constant environment required for normal brain function i.e., neuronal homeostasis. Under normal physiological conditions, the presence of continuous strands of TJs between adjacent ECs of brain capillaries significantly prevents transport of polar solutes, macromolecules and cells from the circulation into the brain through the paracellular pathway (Abbott et al., 2010). On the other hand, under pathological conditions, the leakage of intravascular substances through the disrupted BBB to brain parenchyma occurs principally by increased function of transcellular pathway i.e., vesicular transcytosis malfunctioning of various transporters and efflux pumps such as the P-glycoprotein (P-gp) and/or paracellular pathway (the opening of the intercellular TJs) (Kaya and Ahishali, 2011). OPs inactivate the enzyme acetylcholine esterase (AChE), a serine protease that hydrolyzes the neurotransmitter acetylcholine. Much of the brain damage caused by exposure to OPs does not typically occur at the time of the initial lesion, making secondary neuronal damage a major contributor to the neuronal loss. Although the AChE-related toxicity effects on neurons are well studied, the non-AChE-related effects on other CNS cellular components are still clouded in uncertainty. In this study we used PX as the organophosphate compound model. We hypothesized that PX induces a direct damage to BBB and sought to explore the cellular mechanisms underlying alterations in barrier function independent of AChE inhibition. Several *in vivo* studies have shown that BBB Pe is increased after exposure to OPs (Gupta et al., 1999; Song et al., 2004) and specifically to PX. However, chronic OP treatment has also been shown not to have any effects on the BBB Pe in one study (Rakonczay and Papp, 2001). OPs toxicants used in chemical warfare, such as soman and sarin, have been shown to cause a breakdown in the BBB in adult rats (Carpentier et al., 1990; Petrali et al., 1991; Gupta et al., 1999; Abdel-Rahman et al., 2002). There is a debate whether this effect is only seizure-dependent or directly induced by the OPs (Ashani and Catravas, 1981; Carpentier et al., 1990; Song et al., 2004). A number of *in vitro* studies investigating the direct effects of PX on cellular mechanisms were published, mainly on neurons (Yousefpour et al., 2006; Vatanparast et al., 2007; Pomeroy-Black and Ehrich, 2012; Meijer et al., 2014). A few *in vitro* studies regarding the cellular and molecular aspects involved in direct BBB disruption in response to OPs exposure were published so far (Parran et al., 2005; Balbuena et al., 2011; Li and Ehrich, 2013), showing, for example, that exposure of an *in vitro* BBB model to the OP chlorpyrifos (CPF) or to lead and malathion resulted in loss of electrical resistance and TJ proteins. The effect of CPF on claudin-5 and ZO-1 gene expression was transient and reversible (Karami-Mohajeri and Abdollahi, 2013). Cellular damage induced by ROS is cumulatively referred to as oxidative stress (Prins et al., 2014). Previous findings seem to suggest that disturbances in oxidative processes could play an important role in the toxicity of OPs insecticides (Sitkiewicz et al., 1980). For example, CPF-induced apoptosis was involved in mitochondrial dysfunction through the production of ROS in PC12 neuronal cell line (Lee et al., 2012), and results in human salivary gland cells indicated that superoxide but not peroxide were produced upon PX-treatment (Prins et al., 2014). This outcome raises the possibility that oxidative stress is a trigger of cytotoxicity (Jafari et al., 2012).

Our results demonstrate that PX directly affects the BBB *in vitro* by attenuating viability, integrity and junctional mRNA and protein expression and our results suggest that BLEC change their morphology as an induced mechanism for coping with these barrier damaging modifications and show that preventing the induced cell death, *per se*, cannot rescue BBB integrity even when certain compensating mechanism are still induced.

The results obtained from this research might shed light on important BBB related cellular responses toward OPs exposure and in particular on the underlying mechanisms involved in human BBB disruption after exposure to PX. Attenuating or reversing these responses using different compounds should be considered with care since they are not always trivial.

## Materials and Methods

### Materials

Mouse anti Claudin-5 (zy-352500) diluted 1:200 and rabbit anti-ZO-1 (zy-617300) diluted 1:150 were purchased from Invitrogen (United States). Mouse anti P-gp (517310) diluted 1:100 was from Millipore (United States). Goat anti-VE-cadherin antibody (sc-6458) diluted 1:100 was obtained from Santa Cruz Biotechnology (United States). Cy and Alexa Fluor-conjugated secondary antibodies were acquired from Jackson Immunoresearch (United States) and Molecular Probes (United States), respectively, and used for immunocytochemistry. Z-VAD-FMK was obtained from Adooq-bioscience (187389-52-2, United States) and Santa Cruz Biotechnology (sc-3067, United States). Marimastat from Santa Cruz Biotechnology (sc-202223, United States), MG-132 from Promega (G9951, United States), Tempol (4-hydroxy-TEMPO) from Sigma (176141, United States). Cytotoxgreen was obtained from Essen BioScience (United States) and CellROX-green from Molecular Probes (United States). PX-ethyl was purchased from Sigma (United States), according to Sigma safety data sheet safety measures of eyeshields, face shields, full-face respirator and Gloves should be taken. For assessing the BBB response, PX freshly made in ethanol to 400 mM stock solution was immediately diluted in the medium to the desired final concentrations and added to the cell culture. All other reagents applied in this study were used in accordance to the known literature and the supplier’s guidelines.

### Media

Brain-like endothelial cell and pericytes were grown in ECM medium (Sciencell, United States) that was composed as follows: 5% fetal calf serum (Gibco, United States), ECGS supplements and 50 μg/ml gentamicin (Biological industries, Israel).

### Cells

Human CD34+-derived ECs and bovine brain pericytes were obtained from Artois BBB laboratory where their isolation and differentiation were conducted as previously described (Pedroso et al., 2011; Saint-Pol et al., 2012; Cecchelli et al., 2014). Regarding the collection of human umbilical cord blood: infants’ parents signed an informed consent form, in compliance with the French legislation. The protocol was approved by the French Ministry of Higher Education and Research (CODE-COH Number DC2011-1321). All experiments were carried out in accordance with the approved protocol.

For each experiment, the cells were expanded on gelatin (Sigma, United States)-coated dishes in ECM medium. For BLEC monolayer experiments, CD34^+^ ECs were cultured with pericyte-conditioned-medium for 4 days. For co-culture experiments, 5 × 10^4^ brain pericytes were seeded on 12 well gelatin-coated plates (Costar, Corning, United States) and cultured in ECM medium. Human CD34+-derived ECs were seeded at a density of 8 × 10^4^/insert onto the Matrigel-coated (BD Biosciences, United States) Transwell inserts (3401-Costar, Corning, United States). Cells were grown in co-culture for 6–8 days for Pe assay and acquire BBB properties during this period becoming BLECs (BLECs).

### Permeability Assay

Prior to the experiments, HEPES-buffered Ringer’s solution (RHB) was added to empty wells of a 12 well plate (Costar). Filter inserts, containing confluent monolayers of BLECs in RHB, were subsequently placed in the 12 well plate to acclimate for 1 h, after which compound solution containing the fluorescent integrity marker Fluorescein (50 μg/ml; Sigma, United States) was added to the luminal side, and then placed on a shaker at 37°C. Every 10 min inserts were transferred to a new 12 well plate over a period of 40 min. Aliquots from the abluminal solution were taken from each time point and the fluorescence was quantified. Inserts without cells were tested in each Pe measurement. Fluorescein detection was carried out on an Infinite 200 PRO (Tecan, Switzerland) plate reader using the excitation/emission wavelength (nm) settings: 485/538. Pe coefficient was obtained from the slope of the calculated clearance curve as described in reference (Dehouck et al., 1990). Typical Pe value for the control was Pe = 0.35x10^-3^ cm/min.

### Cell Death by LDH Release

The toxicity of PX was investigated on monolayers of BLEC seeded on 96 well plates or on inserts luminal side and abluminal side in co-culture experiments using the commercially available Cytotoxicity Detection Kit (Promega, United States). An aliquot of 50 μl medium was taken to quantify the lactate dehydrogenase (LDH) release. The test was performed according to the manufacturer’s instructions and absorption was measured at 490 nm by an Elisa plate reader (Tecan, Switzerland).

### Cell Death by Cytotoxgreen Staining

Monolayers of BLECs or bovine brain pericytes were treated with PX in 96 well plates and monitored simultaneously for cytotoxic response kinetics with cytotox green stain (250 nM) for 24 h by live imaging using an IncuCyte imaging system (Essen BioScience, United States). Phase contrast and green fluorescence images were captured every hour and analyzed using the integrated confluence algorithm. Relative cytotoxicity was calculated as the green confluence divided by the phase confluence.

### Viability by MTT Assay

The viability of cells after 24 h of PX treatment was investigated on monolayers of BLEC seeded on 96 well plates, by adding 0.5 mg/ml MTT tetrazole [3-(4,5-dimethylthiazol-2-yl)-2,5-diphenyltetrazolium bromide] (Sigma, United States) to each well. After 3 h at 37°C, the absorbance was measured at 560/630 nm by an Elisa plate reader (Tecan, Switzerland).

### Quantitative Real-Time PCR

RNA from BLECs monolayers was extracted using NucleoSpin RNA II kit (Macherey-Nagel, United States) according to the manufacturer’s instructions, and cDNA was prepared using high capacity cDNA RT kit (Applied Biosystems, United States) according to the manufacturer’s protocols. Real-time PCR was carried out using Fast SYBRGreen master mix (Applied Biosystems, United States) and processed using Step One Plus (Applied Biosystems, United States). Data were normalized to the housekeeping gene GAPDH. The gene expression levels were calculated using the ΔCt method. Primers used were:

      Occludin: Fw-5′-AGGAACCGAGAGCCAGGT-3′;

      Rev-5′-GGATGAGCAATGCCCTTTAG-3′,

      MDR-1: Fw-5′-CCCATCATTGCAATAGCAGG-3′;

      Rev-5′-GTTCAAACTTCTGCTCCTGA-3′,

      Claudin-5: Fw-5′-CTTCCAGAATGGCAAGAGAGTGA-3′;

      Rev-5′-ACCACTGTTCTCCACTGCTCAGA-3′,

      ZO-1: 5′-TGATCATTCCAGGCACTCG-3′;

      Rev-5′-CTCTTCATCTCTACTCCGGAGACT-3′,

      Glut-1: Fw-5′-GGTTGTGCCATACTCATGACC-3′;

      Rev-5′-CAGATAGGACATCCAGGGTAGC-3′,

      VE-Cadherin: Fw-5′-GGTCCCTGAACGCCCTGGTAA-3′;

      Rev-5′-GGAGTGGAGTATGGAGTTGGAGCA-3′

      GAPDH: Fw-5′-GGCCTCCAAGGAGTAAGACC-3′;

      Rev-5′-AGGGGTCTACATGGCAACTG-3′,

### Immunocytochemistry

CD34+-derived ECs were grown on gelatin coated slides or wells until reached monolayer confluence. After treatments, the cells were fixed with 4% paraformaldehyde (PFA) for 10 min at room temperature and exposed to blocking solution [10% horse serum/ 0.1% triton/ (PBS)] for 1.5 h. The ECs were then incubated with anti ZO-1, anti-VE-cadherin, anti-P-gp or anti-Claudin-5 (overnight, 4°C), washed with PBS/0.1% Tween20 and immunostained with appropriate secondary antibodies (1 h, room temperature). Nuclei were counterstained with Hoechst (1 min). Images were taken with Olympus fluorescence microscope (BX43, Japan) or EVOS FL Cell Imaging System (Thermo Fisher Scientific, United States) with 20× objective. When comparison between different treatments was made, the exact same optical settings were used to avoid any misinterpretation of the results. The brightness of some pictures in this paper was increased artificially to emphasize the pattern of protein expression (**Figure 3B**).

### Nuclei Counting and Cell Area Measurement in Confluent Monolayers

Brain-like endothelial cells monolayers were double labeled with the VE-cadherin antibody and the Hoechst reagent for nuclei, as described in the immunocytochemistry section. This allowed the delineation of the cell borders. For counting the number of cells, three random fields were selected in each well and the nuclei in each field of photomicrographs were automatically counted using the ImageJ software (**Figure 5E**). To analyze the cell area distribution, single cell area was calculated using the ImageJ software. The area of at least 90 cells taken from 9 photomicrographs was measured and the cell area distribution was analyzed (**Figure 4**).

### Oxidative Stress Analysis

Cellular oxidative stress was detected using the cell-permeable fluorogenic probe CellROX (5 μM; Molecular Probes, United States). Monolayers of BLECs were treated with PX in 96 well plates and monitored simultaneously for cytotoxic response kinetics with CellROX stain for 48 h by live imaging using an IncuCyte imaging system (Essen BioScience, United States). The intensity of CellROX fluorescence was calculated and analyzed to quantify the ROS level. Phase contrast and green fluorescence images were captured every hour. Relative CellROX intensity was calculated as the average green object integrated intensity divided by the phase confluence.

### Data Analysis

Results are expressed as the mean ± standard error of the mean (SEM), with at least three repeats. ^∗^*P* < 0.05 is considered significant statistically by Student’s *t*-test when comparing between two groups or Ordinary one way ANOVA with Dunnett’s multiple comparisons test when comparing between three groups or more. Analysis was performed using GraphPad Prism 7.0 software. In **Figure 3**, analysis for the rtPCR results was conducted using Kruskal-Wallis test since the sample size was not big enough to assume normality. In the following data acquisition, analysis of results was performed while investigators were blinded to the data (cell area measurements, nuclei counts, micrographs microscopy picturing, automatic Incucyte analysis).

## Results

### BBB *in vitro* Model

**FIGURE 1:**
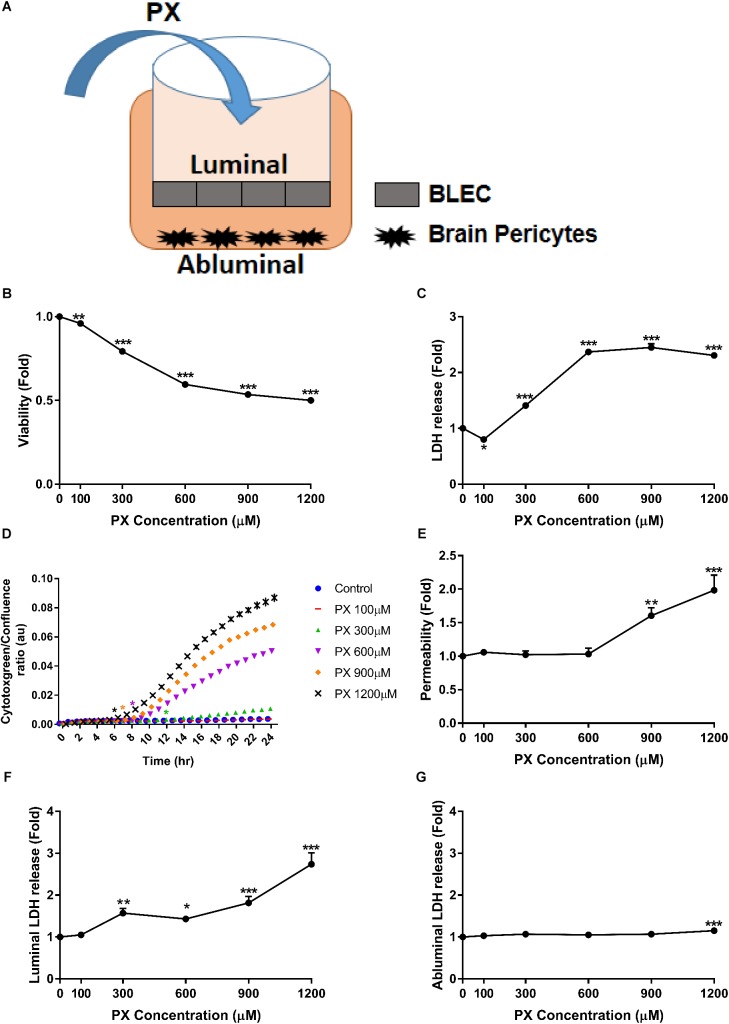
PX dose-dependent increase in toxicity and Pe of the human BBB model. **(A)** Schematic drawing of the *in vitro* BBB co-culture system applied in this study: co–culture model of BLEC grown on matrigel-coated Transwell^®^ permeable inserts with bovine brain pericytes grown at the abluminal side in a non-contact manner, used for functional Pe experiments. **(B–D)** Monolayers of BLECs were treated with PX for 24 h in a dose response experiment. Viability and cell death were examined by **(B)** MTT assay –a representative (out of three) experiment, **(C)** LDH release (*n* = 37-46 from five independent experiments), and **(D)** cytotoxgreen staining (a representative figure, out of three time course independent experiments is shown, *n* = 4–6 for each time point), the relative fraction of dead cells out of the whole population is presented as the ratio of green object confluence to phase confluence, ^∗^marks the first time in which the PX treatment is significantly different from control according to plot color, respectively. **(E,F)** Co-culture BBB *in vitro* model was treated with PX at the luminal side for 24 h in a dose response experiment. **(E)** To assess TJ functionality, Pe of sodium fluorescein (NaF) across the BBB *in vitro* model (from luminal to abluminal side) was assessed (*n* = 11–25 from four independent experiments) and cell death was examined by LDH release at the luminal **(F)** and abluminal **(G)** side (*n* = 11–25 from four independent experiments). Data presented as mean ± SEM. ^∗^*p* < 0.05, ^∗∗^*p* < 0.01 and ^∗∗∗^*p* < 0.001 vs. control (Ordinary one way ANOVA with Dunnett’s multiple comparisons test).

In the current study, to facilitate translation of our results to human studies, we chose to employ the human BBB model developed in the Artois BBB laboratories (Cecchelli et al., 2014). The generation of these cells relies on biological principles observed in the repair of BBB in the human body. The *in vivo* repair of the endothelium is mediated by endothelial progenitor cells that migrate to the sites of endothelial injury, incorporate in the endothelium and differentiate into ECs (Gulati et al., 2003; He et al., 2004). This human BBB model was generated using human cord blood-derived hematopoietic stem cells. CD34^+^ cells were isolated and initially differentiated into ECs followed by the induction of BBB properties by co-culture with brain pericytes. The BLECs express TJ proteins and transporters typically observed in brain endothelium and maintain expression of most *in vivo* BBB properties for at least 20 days (Cecchelli et al., 2014). We used co-culture systems as shown in a schematic representation of this model in **Figure 1A**, and CD34^+^ derived BLEC monolayers cultured with brain pericytes conditioned medium where indicated. PX concentrations at the micromolar range, which are particularly relevant to non-AChE-related cellular responses, were chosen according to similar concentrations used in the literature in neuronal and non-neuronal cells (Carlson and Ehrich, 1999; Carlson et al., 2000; Guizzetti et al., 2005; Massicotte et al., 2005).

### PX-Induced Increase in the Permeability of the BBB Is Only Partially Paralleled With Cell Death

In order to assess the nature of BBB dysfunction imposed by PX, we first measured the direct impact of PX on BBB functionality by Pe and toxicity measurements, using the BBB *in vitro* model exposed for 24 h to PX. PX toxicity profile toward BLEC monolayers is shown in **Figures 1B–D**. A meaningful dose- dependent reduction in viability was observed starting at 300 μM PX of 20.7 ± 0.6 %, 40.4 ± 0.5% for 600 μM, 46.5 ± 0.7% for 900 μM and 50 ± 0.6% for 1200 μM. A cell death measurement by LDH release showed a significant elevation of 1.41 ± 0.04 fold compared to control at 300 μM PX, 2.37 ± 0.05 fold for 600 μM, 2.45 ± 0.07 fold for 900 μM and 2.3 ± 0.05 fold for 1200 μM (**Figure 1C**). In order to assess the initial time point in which PX starts to induce cell death and because LDH release can be also influenced by LDH regulation itself (Valvona et al., 2016), we verified BLEC cell death also by monitoring the cytotoxic response kinetics with cytotoxgreen stain through live imaging. As can be seen in **Figure 1D**, elevation in cell death starts as early as 6 h after initiation of PX treatment for 1200 μM and after 12 h for 300 μM PX.

These concentrations are physiologically relevant since it was proposed based on pharmacokinetic models and biomonitoring data that *in vitro* OPs concentrations higher than 100 μM reflect acute accidental intoxication (Buratti et al., 2007). To evaluate the effect of PX on the BBB paracellular pathway we examined the Pe of the co-culture BBB model after exposure to PX. Adding PX to the luminal side of the BBB model for 24 h increased the Pe to the small non-permeable molecule sodium fluorescein (NaF, MW 376) only at PX concentrations of 900 μM and 1200 μM, by 60.5 ± 12% and 98.3 ± 23%, respectively (**Figure 1E**). This is in spite of the significant toxicity observed in this setting at the luminal side (representing BLEC cell death) already at a much lower concentration of 300 μM PX (**Figure 1F**), which was also observed in the mono-culture setting (**Figure 1C**). These results suggest that BLEC maintain barrier properties (at least partially) even at toxic levels of PX (300 and 600 μM). The LDH release elevation observed at the abluminal side starts with 1200 μM PX (**Figure 1G**) and is of a much lower magnitude than the luminal side. Indeed, the luminal side is immediately exposed to PX and the PX exposure of the abluminal side is secondary. Pericytes cell death may explain this difference.

**FIGURE 2:**
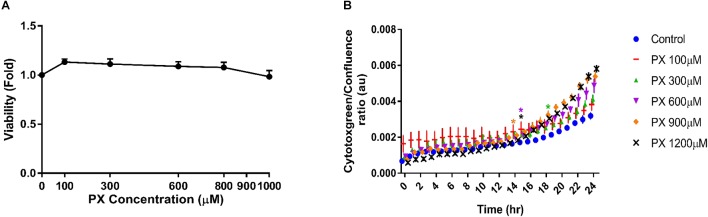
Pericytes show high resilience to PX in comparison to BLEC. Pericytes were treated as mono culture with PX for 24 h in a dose response experiment and **(A)** cell viability was examined by MTT assay (*n* = 13–22 from three independent experiments) and **(B)** by time course experiments of cytotoxgreen staining (a representative figure, out of three independent experiments is shown, *n* = 3–6 for each time point). The relative fraction of dead cells out of the whole population is presented as the ratio of green object confluence to phase confluence, ^∗^marks the first time in which the PX treatment is significantly different from control according to plot color. Data presented as mean ± SEM. ^∗^*p* < 0.05 vs. control. (Ordinary one way ANOVA with Dunnett’s multiple comparisons test).

Next we examined the toxicity of PX on brain pericytes alone as they contribute to BBB formation and maintenance. We found that pericytes are substantially more resistant to PX-induced toxicity than BLEC, displaying no reduction in viability at all PX concentrations tested (**Figure 2A**). Using the cytotoxgreen live imaging assay we observed that PX induces a small dose dependent elevation in pericyte cell death (**Figure 2B**) which is in line with the abluminal LDH release results (**Figure 1G**).

### PX-Induced Alterations in Tight Junctions, Adherens Junction and Transporters Levels

**FIGURE 3:**
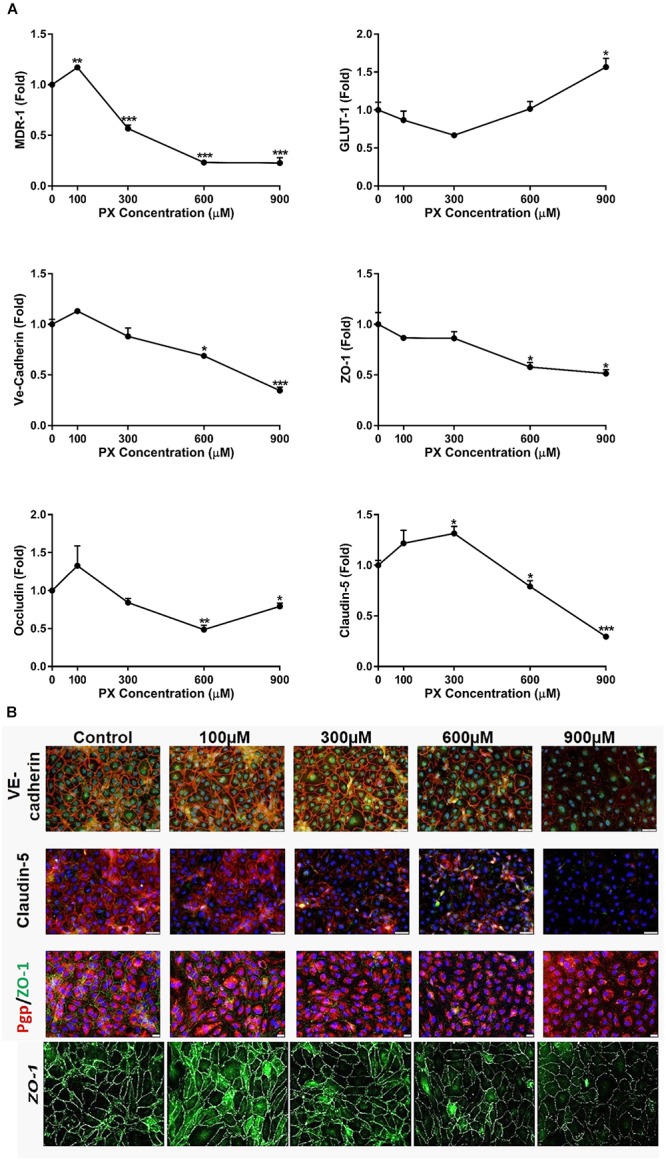
BBB transporters, tight and adherens junction mRNA levels and protein expression are attenuated upon PX exposure. **(A)** Monolayers of BLECs were treated with PX for 24 h and the mRNA expression levels of the junctional genes Occludin, Claudin-5, ZO-1, Ve-Cadherin, and transporter genes Glut-1 and MDR-1 were examined (*n* = 3 with 8–9 technical repeats). **(B)** ECs monolayers were treated with PX for 24 h and protein expression levels and patterns of Claudin-5, ZO-1, Ve-Cadherin and P-gp were examined using immunocytochemistry, one representative picture is displayed. Bar scales for two top panels equal 50 and 20 μm for the third row. Magnification level for the bottom panels is x200. Data presented as mean ± SEM. ^∗^*p* < 0.05, ^∗∗^*p* < 0.01, and ^∗∗∗^*p* < 0.001 vs. control (Kruskal-Wallis one way ANOVA with Dunnett’s multiple comparisons test).

The paracellular Pe of the BBB is closely associated with the expression and function of TJ and AJ (Kniesel and Wolburg, 2000; Nitta et al., 2003). The mRNA levels of different TJ and AJ were measured after 24 h treatment with PX. At the mRNA level, the treatment with PX (**Figure 3A**) resulted in a significant decrease of the mRNA levels for Claudin-5, Occludin, ZO-1, VE-cadherin as well as with one of the major transporters lining the BBB, P-gp (MDR-1), at 600 and 900 μM. Glucose transporter-1 (Glut-1), on the other hand, was elevated at 900 μM PX. After 24 h treatment with 100 μM PX, most TJ, AJ and P-gp mRNA levels showed an increase (statistically significant only for P-gp), implying that at a low PX dosage there is an induction of typical BLEC genes expression in order to maintain barrier properties.

Assessment of proteins expression levels and patterns after EC exposure to PX using immunocytochemistry is displayed in **Figure 3B**. With increasing concentrations of PX, ZO-1, VE-cadherin and Claudin-5 immunostaining is weaker and thinner, evident especially at 900 μM PX where the pattern of protein expression becomes scattered. These results are in accordance with the mRNA levels results. A decrease in the expression at the protein level was observed in parallel to an increase in Pe at 900 μm. These reductions in TJ/AJ protein levels at 900 μM PX may also explain the elevation in Pe at these PX concentrations.

Changes in the patterns of expression and general changes in cell morphology were observed already at lower concentrations, however, at 300 and 600 μM barrier integrity remained intact.

Protein synthesis is clearly essential for the maintenance of the TJs between brain ECs according to data showing that TEER decreased to background values after an overnight treatment with cycloheximide-a protein synthesis inhibitor (Gaillard et al., 2003). Regarding TJ protein degradation, there are a few enzymatic pathways known to be responsible. Among them, 26S proteasome and metalloproteinases (MMPs) degradation of TJ proteins were found (Li et al., 2015). In order to evaluate these enzymatic degradation pathways on the BBB integrity that relies on TJ functionality, we performed Pe experiments using the proteasome inhibitor MG-132 and the pan- MMP inhibitor marimastat (at a concentration of 0.1 μm). We found no improvement in the induced Pe caused by 900 μM PX, while no further cytotoxicity was observed with the addition of the inhibitors by LDH release (data not shown). This implies that these TJ degradation pathways are not primarily involved in the PX induced TJ downregulation.

### Cell Area Enlargement as a Compensatory Mechanism to Maintain Barrier Function After Exposure to PX

Since the increase in the Pe started only at 900 μM PX (**Figure 1E**) while significant toxicity was observed already at 300 μM (**Figures 1C,F**), we speculated that cellular compensatory mechanisms exist to maintain barrier properties despite an obvious cell death under these toxic concentrations. Since viability of BLEC drop at 300 μM and 600 μM, the addition of new cells to close the gap created by dying cells is less probable, hence, we hypothesized that the remaining cells enlarged their territory in order to compensate for the cell loss.

**FIGURE 4:**
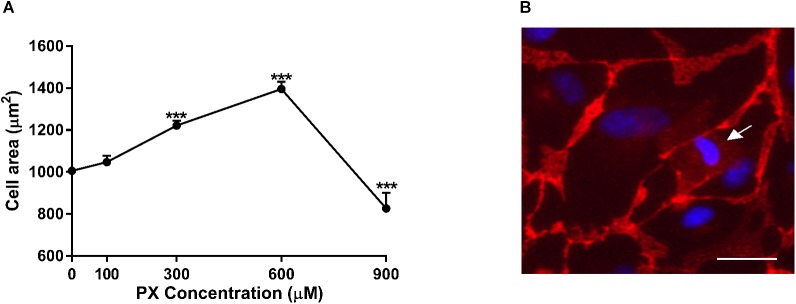
Cell area enlargement as an endothelium compensatory mechanism to maintain barrier properties by PX. **(A)** Dose-dependent cell area expansion in monolayers of BLECs treated for 24 h with PX calculated with imageJ from TJ-immunostained BLECs from at least 9 micrographs (*n* = 146–170 cells per treatment from three independent experiments). **(B)** A dying BLEC (marked with an arrow) covered by neighboring cells with functional adherens junctions at 300 μM PX (immunostaining of VE-cadherin is shown). Data presented as mean ± SEM. ^∗∗∗^*p* < 0.001 vs. control (Ordinary one way ANOVA with Dunnett’s multiple comparisons test). Bar scale equal 20 μm.

To answer this question, we measured the individual cell area of at least 90 cells per treatment. The calculated average cell area (μm^2^) in PX-treated and untreated monolayers is shown in **Figure 4A**. Cell area enlargement of 21.4% started at 300 μM and reached a maximum at 600 μM corresponding to 38.7% increase in cell area and declined by 18% at 900 μM compared to control. The population median was also shifted from 934 μm^2^ for control to 1246 μm^2^ after PX treatment at 600 μM and reduced to 731 μm^2^ at 900 μm. These results may support our hypothesis that the PX-induced cell death was compensated by the expansion of the territory of the remaining cells as a cellular compensatory mechanisms to maintain barrier integrity. A dying BLEC covered by neighboring cells with seemingly functional junctional proteins is shown in **Figure 4B**.

At higher PX concentrations (900 μM) where Pe is increased- cell area declines suggesting the existence of larger amount of dying/dead cells in the culture which are smaller than normal cells and cannot be replaced and covered by the enlargement of adjacent cells.

### Inhibition of Caspases Results in Full Recovery at the Viability Level but Not at the Functionality Level

**FIGURE 5:**
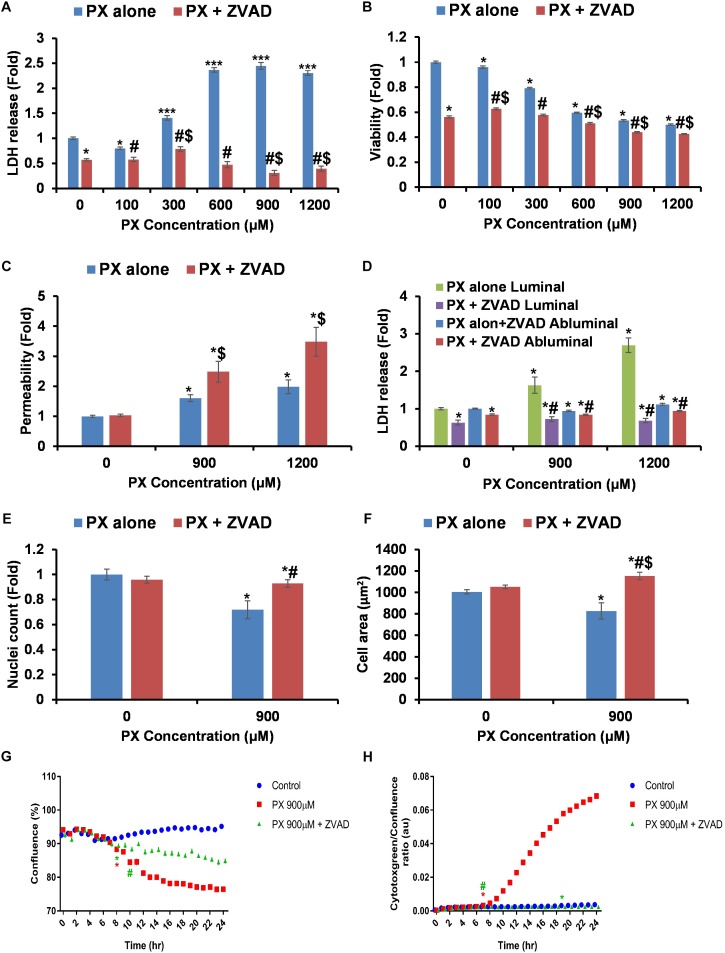
Inhibition of caspases blocks cell death but does not restore the functionality of the barrier. **(A,B)** LDH release (*n* = 12–46 from four independent experiments) **(A)** and MTT assay (*n* = 11–20 from three independent experiments) **(B)** after 24 h PX treatment with ZVAD in a BLEC monolayer. **(C)** Co-cultures of BBB *in vitro* models were treated at the luminal side with PX + ZVAD for 24 h in a dose response experiment. Pe of NaF from luminal to abluminal side was measured (*n* = 12 Transwell inserts from three independent experiments). **(D)** LDH release at the luminal and abluminal sides of the co-culture BBB system was measured to assess cell death. **(E)** Nuclei number was counted with imageJ from at least six micrographs of BLEC monolayers treated with PX ± ZVAD for 24 h. **(F)** Cell area of BLEC monolayers treated for 24 h with PX ± ZVAD calculated with imageJ from AJ-immunostained BLECs micrographs (*n* = 117–190 cells per treatment from three independent experiments). **(G,H)** A representative time course experiment of BLEC monolayers treated for 24 h with PX ± ZVAD, phase contrast confluence is presented (%) **(G)** and cytotoxgreen staining is presented **(H)**, the relative fraction of dead cells out of the whole population is presented as the ratio of green object confluence to phase confluence (a representative figure, out of three time course independent experiments is shown, *n* = 6 for each time point). ^∗^Marks the first time in which the PX treatment is significantly different from control according to plot color. Data presented as mean ± SEM. ^∗^*p* < 0.05 and ^∗∗∗^*p* < 0.05 vs. control, ^$^*p* < 0.05 vs. control+ZVAD and ^#^*p* < 0.05 vs. PX alone at the same concentration, respectively. All results without ZVAD in this figure were taken from the previous figures in this article.

In an effort to find a way to intervene and counteract PX deleterious effects, we attempted to inhibit the death mechanism induced by PX. Previous findings in SH-SY5Y cells showed that PX at 1000 μM induced significant caspase-3 dependent apoptosis (Carlson et al., 2000). To validate induction of apoptosis and to explore a way to repair the BBB endothelium, we used the pan-caspase inhibitor ZVAD-FMK (ZVAD) simultaneously with PX treatment. ZVAD restored most of PX-induced alterations in BLEC: cell death was fully blocked using 50 μM ZVAD (**Figure 5A**), a decrease in viability started only at 600 μM and not at 300 μM with ZVAD (**Figure 5B**) and to a much smaller effect (**Figure 1A**): reduction of 40.4 ± 0.5% for 600 μM vs. 0 μM compared to 9 ± 1% for 600 μM+ZVAD vs. 0 μM+ZVAD. Surprisingly, Pe was not restored to normal for 900 and 1200 μM, and was even elevated by ZVAD at these PX doses (**Figure 5C**), despite the complete inhibition of cell death gained with ZVAD in the same co-culture system (**Figure 5D**). Importantly, ZVAD by itself (0 μM+ZVAD) did not elevate Pe. To ensure that cell number was indeed elevated by ZVAD we counted nuclei and show that nuclei number at 900 μM+ZVAD was slightly but significantly elevated (7% difference compared to normal) (**Figure 5E**). In order to understand why BBB integrity was not restored by ZVAD and was even more compromised, we measured cell area and found that it was elevated for 900 μM with the addition of ZVAD even to a higher level than control (**Figure 5F**). By on-line phase confluence measurement which assesses both cell number and cell area parameters, one can see that ZVAD treatment on 900 μM PX indeed improved the monolayer cell coverage as early as 10 h after PX treatment initiation (**Figure 5G**). In addition, rescue of cytotoxicity by ZVAD is also seen very early, at 7 h after PX treatment initiation (**Figure 5H**). In summary, a combined treatment with PX and ZVAD resulted in elevated Pe to a higher extent than PX alone. Despite the clear inhibition of cell death and cell area enlargement by ZVAD at 900 μM PX, these parameters were not able to restore barrier integrity. Nuclei number was not fully recovered but this result alone could not explain an even greater increase in Pe which is shown with the addition of ZVAD.

### PX Induces Reactive Oxygen Species Formation

**FIGURE 6:**
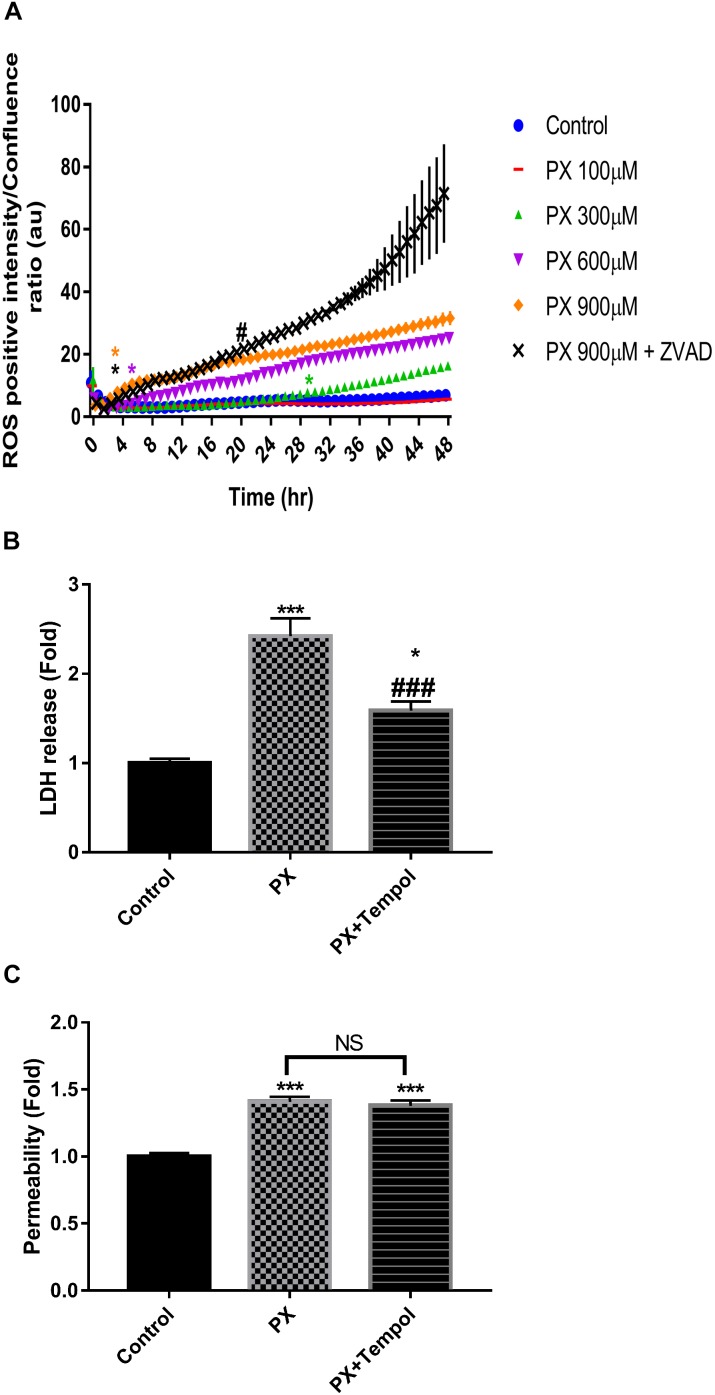
Dose and time-dependent increase in ROS levels by PX is not reversed by ZVAD. Monolayers of BLECs were treated with PX for 48 h in a dose response experiment. **(A)** A representative time course experiment of CellRox green staining, the fluorescence intensity of ROS-positive cells normalized to the whole population is presented as the ratio of green object integrated intensity to phase confluence (a representative figure, out of three time course independent experiments is shown, *n* = 6 for each time point). ^∗^Marks the first time in which the PX treatment is significantly different from control, #marks the first time in which the PX treatment is significantly different from 900 μM PX; after 38 h 900 μM PX+ZVAD is not significantly different from 900 μM PX according to plot color. ^∗^*p* < 0.05 vs. Control, #*p* < 0.05 vs. PX at 900 μM. **(B)** Co-cultures of BBB *in vitro* models were treated at the luminal side with 900 μM PX with or without TEMPOL at 150 μM for 24 h. LDH release at the luminal side of the co-culture BBB system was measured. **(C)** Pe of NaF from luminal to abluminal side was measured (*n* = 9–12 Transwell inserts from 3 independent experiments). Data presented as mean ± SEM. ^∗^*p* < 0.05 and ^∗∗∗^*p* < 0.001 vs. control, ^###^*p* < 0.001 vs. PX alone (Ordinary one way ANOVA with Dunnett’s multiple comparisons test). NS, no statistical differences.

It has been previously shown that ROS can affect TJs integrity (Musch et al., 2006). To determine if PX induces ROS formation and subsequent oxidative stress, BLEC were analyzed for the presence of ROS in time and dose response experiments. As can be seen in **Figure 6**, following ROS production for 48 h, a dose response elevation is observed. For 900 μM PX, a significant elevation in ROS levels is seen as early as 3 h after the initiation of PX treatment. In microvascular ECs, increased ROS formation leads to caspase-3 activation following ischemia reperfusion injury and a caspase-3 inhibitor significantly attenuated the induced hyperpermeability and protected TJ integrity (Alluri et al., 2014). Here, not only that the addition of the pan-caspase inhibitor ZVAD to 900 μM PX treatment did not normalize ROS levels but it elevated them above the 900 μM PX levels before the 24 h time point. This could provide an explanation to the increase in Pe seen with ZVAD at 900 μM PX (**Figure 5C**). Next, we carried out a Pe experiment with a ROS scavenger (**Figures 6B,C**). We chose to treat the BBB model with Tempol which is known as a mimetic of superoxide dismutase and is considered a general-purpose redox-cycling agent. In another study, tempol provided almost full protection in a viability test of microglial cells treated with the OP dichlorvos and was found to significantly decrease ROS production induced by dichlorvos (Sunkaria et al., 2014). As can be seen in **Figure 6B**, rescue of cell death by tempol was partial, but it did not rescue the PX-induced Pe (**Figure 6C**). We conclude that ROS is the cause, at least in part of the cell death induced by PX but inhibiting cell death, as was seen with ZVAD is not able to rescue BBB integrity. Next, we carried out a Pe experiment with a ROS scavenger (**Figures 6B,C**). We chose to treat the BBB model with Tempol which is known as a mimetic of superoxide dismutase and is considered a general-purpose redox-cycling agent. In another study, tempol provided almost full protection in a viability test of microglial cells treated with the OP dichlorvos and was found to significantly decrease ROS production induced by dichlorvos (Sunkaria et al., 2014). As can be seen in **Figure 6B**, rescue of cell death by tempol was partial, but it did not rescue the PX-induced Pe (**Figure 6C**). We conclude that ROS is the cause, at least in part of the cell death induced by PX but inhibiting cell death, as was seen with ZVAD is not able to rescue BBB integrity.

## Discussion

Our main objective was to understand the BBB cellular response toward the organophosphate PX. We propose a mechanism for BLEC response toward PX that relies on a morphological coping mechanism in which the cells enlarge their cell area at toxic doses of PX in order to avoid gaps formation created by the dying cells. Adding PX to our BBB system for 24 h resulted in caspase dependent cell death which was compensated by enlargement of the cell area in order to maintain an intact barrier. This was paralleled with a decrease in TJ expression resulting in a net elevation in Pe. Inhibition of apoptosis using a pan caspase inhibitor was still accompanied by the cell area enlargement mechanism but barrier integrity was even more compromised.

There was a toxic range (“sub lethal”, ∼300–600 μM) where the Pe remained intact although cells were dying (**Figures 1E,F**), suggesting for a compensatory mechanism of the BBB to keep essential barrier properties under stressful conditions. We showed (**Figure 4**) that the dying cells are quickly covered by adjacent BLEC with functional junctional proteins, not leaving substantial gaps in the monolayer, thus maintaining essential barrier properties.

At PX concentrations where both toxicity and Pe are increased- there is also a meaningful reduction in cell area in comparison to the “sub-lethal” concentrations (**Figure 4A**). Lastly, compared to control, viability was not restored by adding ZVAD to 900 μM PX, and compared to 900 μM PX alone it was even decreased. In this scenario, we suggest an explanation to the increased Pe by ZVAD through BLEC attenuated functionality and hence probable TJ/AJ protein damaged functionality.

It has been previously shown that PX can induce caspase-3 activation at 1000 μM and apoptotic cell death in other cell types like neuroblastoma cells (Carlson et al., 2000) and that inhibition of caspases protected against PX-induced cell death in pulmonary epithelium (Angelini et al., 2015). Caspase-3 contributes to ZO-1 and Claudin-5 tight-junction disruption (Zehendner et al., 2011). In a study done on a rat brain microvascular endothelial cell line, OPs reduced TJ and scaffold proteins levels. Gene expression, however, did not appear to correlate with levels of proteins, indicating that the effects inducing the reduction in TJ protein levels did not follow changes in gene expression but may be post-translational (Balbuena et al., 2011). In our human system, a caspase-dependent-degradation of TJs is less probable since the caspase inhibitor ZVAD did not restore barrier integrity and even elevated Pe (**Figure 5C**), but recovery of TJ protein levels by inhibition of caspases could be masked by additional barrier damaging effects. Examining involvement of the 26 S proteasome and MMPs in TJ degradation also displayed no significant effects in rescue of the BBB integrity damaged by PX (data not shown).

On the molecular level, OPs toxicants target and bind proteins, including serine, tyrosine, lysine and histidine residues and can disrupt their function (Ashbolt and Rydon, 1957; Prendergast et al., 2007; Grigoryan et al., 2008, 2009a,b; Schopfer et al., 2010). For example, tubulin is readily labeled by OPs compounds (Grigoryan et al., 2008, 2009b; Jiang et al., 2010). Alterations in tubulin polymerization induce the activity of multiple pro-apoptotic proteins (Giacca, 2005), suggesting a route by which OPs can induce apoptotic cell death.

It was previously shown that OPs compounds dampen the rate of protein synthesis (Harvey and Sharma, 1980; Rodriguez et al., 2006) and this may also explain the reduction in TJ protein levels with PX after 24 h. On the other hand, significant increase in total protein were observed following exposure to 100 μM parathion but not to PX in neuroblastoma cells suggesting that PX may not affect protein synthesis and it depends on its concentration (Carlson and Ehrich, 2001).

Regarding mRNA levels, it was previously reported that treating human lymphocytes with the OP malathion at low concentrations resulted in an increase of RNA synthesis, while, higher concentrations of malathion inhibited nucleic acid synthesis depending on the dose and the time of introduction (Czajkowska and Walter, 1980). This may explain our observation of a trend of TJ mRNA increased levels at 100μM PX after 24 h (**Figure 3A**) and the reduction at 900μM PX. However, this is probably not a general mechanism since Glut-1 mRNA was elevated at 900 μM PX. This may imply a higher brain energy demand when OPs are circulating in the blood and the BBB responds in elevating Glut-1 levels. There is a previously reported study which might explain Glut-1 mRNA induction. In this study the cytotoxic factor tumor necrosis factor-α (TNFα) increased the abundance of Glut-1 transcripts in brain ECs (Boado et al., 1994). Another report has shown that dichlorvos (an organophosphate) exposure can lead to activation-induced cell death in microglia preceded by increased production of TNF-α (Sunkaria et al., 2014). Speculatively, TNFα could play a role in PX induced cytotoxicity and induce upregulation of Glut-1 mRNA. In this context, learning the transcriptional regulation modulated by PX is of great importance and should be further examined in future studies.

Different assays to assess cell viability/death may result in diverse outcomes. The cell death assays examine the integrity of the cell membrane, which can be measured by the cytoplasmic enzyme activity LDH released by damaged cells or by cytotoxgreen staining while the MTT viability assay measures the metabolic activity of the mitochondria. The MTT assay does not always correctly quantify cell protective effects, which likely reflects differences in the point of the death pathway that a protective agent acts on. Therefore, it may provide information regarding whether specific anti-apoptotic agents act up or downstream of mitochondrial dysfunction (Lobner, 2000). The differences between MTT and LDH assays in the level of ZVAD protection (**Figures 5A,B**) suggest that ZVAD acts downstream of mitochondrial dysfunction in the case of PX. Indeed, PX is known to induce apoptosis in other cell types via activation of mitochondrial pathways (Saleh et al., 2003).

Nuclei number is dependent on cell death and proliferation. Comparing the nuclei number data (**Figure 5E**) to the viability measurement by MTT (**Figure 5B**), a bigger amplitude in the reduction of viability is seen compared to the reduction in nuclei number. This may imply that PX affects other cellular parameters which in turn influence the viability of the remaining cells in culture. ZVAD alone shows reduction in MTT and LDH release (**Figures 5A,B**), which could lead to the assumption that cell number should be decreased by ZVAD itself, but according to our nuclei count (**Figure 5E**) which is not changed by ZVAD in the control cells, this is not the issue at hand.

Treatment with 900 μM PX resulted in ROS level elevation after a short exposure time (**Figure 6A**). A short time after that, cytotoxicity was induced (**Figure 5H**). Although the addition of ZVAD completely reduced the PX-induced cytotoxicity (**Figure 5H**) it initiated a higher induction of oxidative stress at a later time point that may ultimately lead to the increase in barrier Pe. Approximately 19 h after the addition of PX and ZVAD, an induction of a higher oxidative stress compared to 900 μM PX alone is seen (**Figure 6A**).

A study by Saleh et al. (2003) delineated the sequence of events involved in PX stimulated apoptosis in T-lymphocytes. Their results indicate that PX induces apoptosis by altering mitochondrial transmembrane potential, causing the release of cytochrome c into the cytosol, which leads to activation of the apoptosome. One of the mechanisms suggested for this stimulation is increased production of ROS (Morkunaite-Haimi et al., 2003). The presence of ZVAD in their study did not inhibit PX-induced disruption of the mitochondrial membrane potential. If this is also the case in our cellular model, ROS could be generated upstream to caspase activation. Thus, caspase inhibition would not reduce ROS production as indeed seen in our results where ZVAD even elevated ROS with PX treatment implicating that cells anticipated to die are rescued while the ROS are accumulated. Oxidants not only disrupt perijunctional actin but also cause redistribution of tight junctional proteins, resulting in compromised barriers (Musch et al., 2006). As can be seen in **Figure 6B**, rescue of cell death by the ROS scavenger -tempol was partial, but it did not rescue the PX-induced Pe (**Figure 6C**). This implies that BBB dysfunction isn’t dependent on ROS in this case, therefore, tempol doesn’t save the BBB integrity, TJ damage could be the major cause of loss in BBB integrity so reducing cell death alone doesn’t help. On the other hand, PX induced cell death is dependent on ROS thus tempol decreases its levels. The TJ sealing is intact at 600 μM PX (no Pe elevation, (**Figure 1E**) while cell death is already induced at this PX concentration (**Figure 1F**) and ROS levels have elevated (**Figure 6A**). These results suggest that TJ protein levels and function are less sensitive to ROS production than cell death, and this is why tempol presumably had a diminished effectivity on TJ function. In conclusion, although literature does imply the existence of a downregulation mechanism of ROS on TJ (Musch et al., 2006), in our system ROS have only a minor or no effect on TJ integrity at 900 μM PX. By inhibiting cell death and examining the resulting cellular outcomes we were able to show that reduction of cell death by the pan-caspase inhibitor ZVAD indeed attenuated cell death (**Figure 5D**). In addition, it enabled the endothelial monolayer to stay intact by restoring gap formation due to less cells disappearing (**Figure 5E**) and due to enlargement of their cell area (**Figure 5F**). Surprisingly, not only that the Pe of the barrier was not rescued as a result of these beneficial effects, it was actually elevated (**Figure 5C**). In this line, ROS levels also increased in response to the inhibition of caspases (**Figure 6A**). Overall, inhibiting cell death in order to rescue the barrier integrity by closing the gaps created by dying cells did not fulfill this aim and even worsened the barrier properties and the oxidative stress induced in the monolayer. This suggests that inhibition of cell death *per se* was not successful in rescuing the barrier

A previous *in vivo* study showed that intracerebroventricular (icv) application of ZVAD had attenuated BBB Pe caused by stress conditions of subarachnoid hemorrhage (Park et al., 2004). This is apparently in contrast to our results that show increase in Pe by ZVAD in stress conditions. Here we focused on the net effect of caspase inhibition on *in vitro* BLECs treated with the cell death inducing compound PX in comparison to the *in vivo* study where the gross effect of caspase inhibition on all central nervous system components was examined under stress conditions. Moreover, ZVAD as a peptidic inhibitor is not likely to cross the BBB, so administration of this inhibitor at the luminal side (and not abluminal like in icv injection) resembles a more realistic state where upon PX exposure ZVAD that will be administered will first encounter the luminal BBB endothelium and only if or when the barrier is compromised it will cross to the brain side.

Organophosphorus compounds were shown to induce morphological modifications in cell cultures (Massicotte et al., 2003; Parran et al., 2005). In neuroblastoma cells (Prins et al., 2010) PX exposure resulted in changes in cytoskeletal protein expression. The cellular compensatory mechanism observed in our study is expected to involve the cytoskeleton that participate and governs a lot of these morphological changes. Thus, in future studies we aim to understand the signaling cascades PX induces on BLEC that may cause these changes in order to find ways to manipulate it to our advantage. In addition, since in other stress models Pe was shown to be ROS and caspase-3 dependent and was able to be regulated by their inhibitors (Alluri et al., 2014), in the following study we will attempt to reverse PX effects through inhibition of a number of ROS and specific caspases and not a pan-inhibition that could mask certain beneficial activities. Understanding the BBB multimodal regulation and its ability to selectively ingress OPs is critical in the development of better medical countermeasures to treat OPs toxicity.

## Author Contributions

OR, SEG, DM, YB, RDO, and SL-Z performed the experiments. FG and LD contributed to the development of the BBB model. OR and IC designed the study. OR, MB, FG, and IC drafted the manuscript.

## Conflict of Interest Statement

The authors declare that the research was conducted in the absence of any commercial or financial relationships that could be construed as a potential conflict of interest.

## Abbreviations

AJ: Adherens junction
BBB: blood-brain barrier
BLECs: Brain-like endothelial cells
OPs: organophosphates
Pe: permeability
PX: paraoxon
ROS: reactive oxygen species
TJ: tight junction
ZO-1: Zonula Occludens 1
